# Comment on “The Success of Cataract Surgery and the Preoperative Measurement of Retinal Function by Electrophysiological Techniques”

**DOI:** 10.1155/2015/139306

**Published:** 2015-12-14

**Authors:** Gökhan Özge, Fatih C. Gundogan, Tarkan Mumcuoğlu

**Affiliations:** Department of Ophthalmology, GATA Medical School, Etlik, 06018 Ankara, Turkey

We congratulate An et al. [1] for their study entitled “The Success of Cataract Surgery and the Preoperative Measurement of Retinal Function by Electrophysiological Techniques.” The authors investigated electrophysiologic and clinical data in patients with cataract before and after cataract surgery. The study has a good design and adds valuable data to the literature about objective retinal function in cataract which may be regarded as a public health problem.

We have read the paper with great interest. The authors investigated the differences between many parameters and found statistically significant differences between cataract patients and healthy subjects in some of them. We know that as the number of statistical comparisons between two groups increase, the probability of finding a significant difference by chance also increases. This issue is solved somewhat by Bonferroni correction. We wonder whether the authors used Bonferroni correction in the statistical analyses. If they did not use it, Bonferroni correction may change statistically significant *P* values (values under 0.05) to insignificant range. We suggest that the authors perform the statistical analyses even without categorizing the cataract group into subgroups. The authors may simply compare the cataract group and healthy group in terms of ERG, PVEP, and mfERG parameters.

In Figure 4, we see that amplitude of central point mfERG has a significant correlation with preoperative visual acuity. However, amplitude density of mfERG has insignificant correlation with visual acuity. What do the authors mean by these two variables? Both variables have the same unit, that is, nV/deg^2^.
